# Norcantharidin overcomes vemurafenib resistance in melanoma by inhibiting pentose phosphate pathway and lipogenesis *via* downregulating the mTOR pathway

**DOI:** 10.3389/fphar.2022.906043

**Published:** 2022-08-12

**Authors:** Lei Wang, Wuxiyar Otkur, Aman Wang, Wen Wang, Yitong Lyu, Lei Fang, Xiu Shan, Mingzhou Song, Yan Feng, Yi Zhao, Hai-Long Piao, Huan Qi, Ji-Wei Liu

**Affiliations:** ^1^ Department of Oncology, First Affiliated Hospital of Dalian Medical University, Dalian Medical University, Dalian, China; ^2^ CAS Key Laboratory of Separation Science for Analytical Chemistry, Dalian Institute of Chemical Physics, Chinese Academy of Sciences, Dalian, China; ^3^ Department of Geriatric Oncology, Dalian Friendship Hospital, Dalian, China; ^4^ Department of Thoracic Surgery, Lung Cancer Diagnosis and Treatment Center of Dalian, First Affiliated Hospital of Dalian Medical University, Dalian, China; ^5^ Department of Computer Science, New Mexico State University, Las Cruces, NM, United States; ^6^ Graduate Program in Molecular Biology and Interdisciplinary Life Sciences, New Mexico State University, Las Cruces, NM, United States

**Keywords:** vemurafenib, resistance, norcantharidin, pentose phosphate pathway, lipogenesis, mTOR. Norcantharidin overcomes Vem-resistance in melanoma

## Abstract

Melanoma is the most aggressive type of skin cancer with a high incidence and low survival rate. More than half of melanomas present the activating BRAF mutations, along which V600E mutant represents 70%–90%. Vemurafenib (Vem) is an FDA-approved small-molecule kinase inhibitor that selectively targets activated BRAF V600E and inhibits its activity. However, the majority of patients treated with Vem develop acquired resistance. Hence, this study aims to explore a new treatment strategy to overcome the Vem resistance. Here, we found that a potential anticancer drug norcantharidin (NCTD) displayed a more significant proliferation inhibitory effect against Vem-resistant melanoma cells (A375R) than the parental melanoma cells (A375), which promised to be a therapeutic agent against BRAF V600E-mutated and acquired Vem-resistant melanoma. The metabolomics analysis showed that NCTD could, especially reverse the upregulation of pentose phosphate pathway and lipogenesis resulting from the Vem resistance. In addition, the transcriptomic analysis showed a dramatical downregulation in genes related to lipid metabolism and mammalian target of the rapamycin (mTOR) signaling pathway in A375R cells, but not in A375 cells, upon NCTD treatment. Moreover, NCTD upregulated butyrophilin (BTN) family genes, which played important roles in modulating T-cell response. Consistently, we found that Vem resistance led to an obvious elevation of the p-mTOR expression, which could be remarkably reduced by NCTD treatment. Taken together, NCTD may serve as a promising therapeutic option to resolve the problem of Vem resistance and to improve patient outcomes by combining with immunomodulatory therapy.

## Introduction

Melanoma is derived from malignant melanocytes, which promote migration and metastasis due to neural crest origin. Despite accounting for only 1% of skin cancer, it constitutes about 80% of deaths from all, with a 5-year survival rate of between 5% and 19% (Sandru et al., 2014). BRAF mutated melanoma accounts for over 50% of melanoma, of which V600E mutant represents 70%–90% (Davies et al., 2002). Vemurafenib (Vem) is an FDA-approved drug for the treatment of late-stage melanoma. By specifically targeting V600E-mutated BRAF, Vem has shown appreciable antitumor activity, and improved survival rates in patients with BRAF V600E-mutant melanoma (da Rocha Dias et al., 2013). Unfortunately, most of these patients treated with Vem will develop acquired resistance, which raises subsequent questions on its underlying mechanism, and urges for a counteract strategy.

Widely acknowledged molecular mechanisms of resistance against BRAF inhibitors include reactivation of the mitogen-activated protein kinase (MAPK) pathway and the phosphoinositide 3-kinase (PI3K)-AKT pathway, secondary MAPK/ERK mutations, and phenotypic alterations (Luebker and Koepsell, 2019; Tangella et al., 2021). Much worse, the acquired resistance is almost inevitable. Most recently, the contribution of metabolic rewiring to drug resistance has become appealing (Cantor and Sabatini, 2012). BRAF V600E mutated melanoma cells reprogrammed their metabolism compared to parental cells through various mechanisms (Hall et al., 2013; Vazquez et al., 2013; Hernandez-Davies et al., 2015). These metabolic alterations derive cancer cells to develop new metabolites addictions and also account for the development of drug resistance. However, such metabolic pathways also represent the vulnerability of drug-resistant cells. Herein, we proposed to apply a potential anticancer drug that exhibits metabolic modulation property as a novel strategy to overcome the Vem resistance.

Several clinical anticancer drugs have been documented to mediate cancer metabolism. Hence, combining Vem with such agents may provide new insights to overcome the resistance of Vem to BRAF mutated melanoma. NCTD is a potent anticancer drug with low toxicity in clinical application (Tarleton et al., 2012; Gao et al., 2017). A variety of studies have proved that NCTD exhibited a powerful and extensive antitumor effect on many types of cancer, such as leukemia (Yi et al., 1991), cholangiocarcinoma (Wang et al., 2020), nasopharyngeal carcinoma (Chen et al., 2018), lung cancer (Luan et al., 2010), and liver cancer (Li et al., 2010). The antitumor mechanisms of NCTD mainly include suppressing protein phosphatase (Chen et al., 2017), inducing apoptosis (Lin et al., 2017; Wu et al., 2018) as well as modulating Wnt/beta catenin signal (Wang et al., 2015; Xie et al., 2016), the AMPK pathway (Chen et al., 2017), and the c-Met/EGFR pathway (Qiu et al., 2017). Previous studies also found that NCTD could induce alterations in lipid metabolism of hepatocytes (Zhao et al., 2019), and also suppressed glycolysis in colorectal cancer cells (Zhang et al., 2020), implying its effect on modulating cancer cell metabolism. Thus, NCTD became a promising candidate agent for our proposal.

In the present study, the cytotoxicity assays of the aforementioned proliferation suppression agents were performed in Vem-resistant melanoma cells (A375R) and parental melanoma cells (A375). We found that NCTD exhibited more significant effects of inhibiting the proliferation against A375R than A375. Furthermore, we employed metabolomics and lipidomics analyses as well as an RNA sequencing platform in A375R and A375 cells with or without NCTD treatment to discover the potentially targeted metabolism-gene network. Our results indicate that NCTD may serve as a promising therapeutic agent to overcome the Vem resistance, and reveal targetable metabolic pathways that may account for the Vem resistance and drug action.

## Materials and methods

### Cell lines and cell culture

Human melanoma cells A375 (Stem Cell Bank, Chinese Academy of Sciences) were maintained in a DMEM medium (Gibco, United States) by adding 10% fetal bovine serum (FBS) (Gibco, United States), 1% penicillin–streptomycin (100 µ/ml penicillin and 100 μg/ml streptomycin), and 5% CO_2_ in an incubator at 37°C.

Vem (PLX4032) was purchased from Selleckchem (Houston, TX, United States), and A375 cells were cultured in the DMEM medium supplemented with Vem from 0.05 gradually up to 10 μM in 3 months to establish drug-resistant cells (A375R) as the previous study described (Carpenter et al., 2019). A375R cells were maintained in the presence of 2 µM vemurafenib to keep the characteristics.

### Cell counting kit-8 assays

A375 and A375R cells were seeded at a density of 3 × 10^3^ and 4.5 × 10^3^ cells per well, respectively, in 96-well plates. Twenty-four hours later, Vem at concentrations from 0 to 2.5 μM for A375 and 0–80 μM for A375R, cisplatin (Selleckchem, United States) with the concentration from 0 to 40 μM, 5-fluorouracil (5-Fu) (Selleckchem, United States) with the concentration from 0 to 50 mg/ml, bufalin (TargetMol, United States) with the concentration from 0 to 200 nM, IMD-0354 (Selleckchem, United States) with the concentration from 0 to 10 μM, and NCTD (TargetMol, United States) at concentrations from 0 to 400 μM were added to the medium for 48 h of treatment. Briefly, cell viability was analyzed by Cytation 5 (BioTek, United States) at 450 nm after using the Cell counting kit-8 (HY-K0301, MedChemExpress) for 2 h of incubation, and the 50% inhibitory concentrations (IC_50_) values were calculated.

### Colony formation assays

The crystal violet staining method was used to perform Cell proliferation assays. Briefly, 5 × 10^3^ A375 cells and 1 × 10^4^ A375R cells were plated in each well in 12-well plates and treated with NCTD (0, 25, 50, and 75 μM), and incubated for 7 days. Next, the cells were stained with 0.1% crystal violet. Finally, the viable cells were imaged by an imaging system (Tanon, United States).

### Cell cycle assays

A375 and A375R were treated with different concentrations (0, 25, 50, and 75 μM) of NCTD for 12 h, then harvested and washed once with PBS. Following centrifugation, the cells were fixed with 70% ethanol at 4°C overnight. On the next day, each sample was stained with 25 μl PI and 10 μl RNase A at room temperature for 30 min according to the manufacturer’s protocols (Beyotime, China). Each sample was detected using a flow Cell Sorter (Sony, SH8005, Japan) and analyzed by using Cell Sorter Software ver 2.1.3.

### Apoptosis assays

A375 and A375R were treated with different concentrations (0, 25, 50, and 75 μM) of NCTD for 24 h, then harvested and washed once with PBS. The apoptosis analysis was evaluated using the Annexin V-FITC/PI Apoptosis Detection kit according to the manufacturer’s protocols (Solarbio Science, China). Each sample was detected using a flow Cell Sorter (Sony, SH8005, Japan) and analyzed by using Cell Sorter Software ver 2.1.3.

### Western blotting

The cells were lysed in RIPA buffer containing protease inhibitors and phosphatase inhibitors, then the BCA kit (TIANGEN BIOTECH, PA115) was used for determining the total protein concentration. Subsequently, the same amounts of proteins were separated by 8%–10% sodium dodecyl sulfate-polyacrylamide gel electrophoresis (SDS-PAGE) and they were blotted onto PVDF membranes (Bio-Rad, Hercules, CA, United States). Membranes were incubated with primary antibodies against human Cyclin D1 (CST, 55506T), c-Myc (Proteintech, 10828-1-AP), Cyclin B1 (Abcam, ab32053), P-glycoprotein (Abcam, ab170904), ERK1/2 (Abcam, ab184699), p-ERK (Abcam, ab201015), p-AKT S473 (CST, 3787S), p-mTOR (CST, 2971S), m-TOR (Abcam, ab32028), and Vinculin (Proteintech, 66305-1-lg) at 4°C overnight. Then, they were maintained in horseradish peroxidase-conjugated secondary antibodies (Jackson ImmunoResearch, AB_2313567; Jackson ImmunoResearch, AB_10015289) for 1.5 h at room temperature. The protein bands were incubated with chemiluminescence detection reagents (Thermo Fisher Scientific) and visualized with the Tanon-5200 chemiluminescent imaging system (Tanon). Protein levels were analyzed by imageJ software (version 1.47v; national institutes of Health) with vinculin as the loading control.

### RNA sequencing

TRIzol was used to extract RNA from A375 cells, A375R cells, A375 + NCTD (60 μM) cells, and A375R + NCTD (38 μM) cells. The concentration and purity of total RNA were determined by Cytation 5 (BioTek, United States). The RNA quality was measured by the OD value under 260 nm/280 nm, which was controlled at least 1.8. Total RNA samples were analyzed by using next-generation sequencing on the Illunmina NovaSeq platform (Hangzhou Kaitai Biotechnology Co., Ltd., China).

### Lipid chromatography–mass spectrometry-based metabolomics and lipidomics analyses

Metabolites of four groups including A375, A375R, A375 + NCTD, and A375R + NCTD were extracted by a mixture of methanol and chloroform, then ultrapure water was added. After centrifugation at 15,000 g for 15 min at 4°C, the filtered aqueous phase was freeze-dried in a vacuum concentrator. Subsequently, chromatographic separation was performed by the ACQUITY^™^ Ultra Performance Liquid Chromatography (UPLC) system (Waters, Milford, MA, United States). Afterward, a coupled AB Sciex tripleTOF 5600 plus mass spectrometer (Applied Biosystems Sciex, Foster City, CA, United States) was used for the global metabolomics and lipidomics profiling. The detailed LC-MS analytical methods were conducted as previously described (Hoene et al., 2014).

### Capillary electrophoresis–mass spectrometry-based metabolomics analysis

The analysis of metabolites was performed by using a CE system (G7100A, Agilent, Santa Clara, CA, United States) combing with time of flight (TOF) mass spectrometry (G6224A, Agilent). All samples were separated by the fused silica capillary [i.d. of 50 μm; total length of 80 cm; Human Metabolome Technologies (HMT), Tsuruoka, Japan]. Quantitative Analysis Software (Agilent) was used for peak extraction and identification. The detailed CE-MS analytical methods were conducted as previously described (Zeng et al., 2014).

### Analysis of the metabolome data

We identified significantly changed metabolites on both the CE-MS and LC-MS metabolomics data associated with the application of NCTD to the cell lines. One-way ANOVA between the A375R and A375 sample pair and between A375R + N and A375R was performed using the R programming language linear model function lm(). *p*-values were adjusted for multiple testing by the Benjamini–Hocheberg method to calculate false discovery rates (Benjamini and Hochberg, 1995). A false discovery rate not greater than 0.05 was used to determine statistical significance of changed metabolites. Downregulation of a metabolite is defined by a high concentration in the A375R samples but low in both A375 and A375R + NCTD samples. Upregulation of a metabolite is defined by a low concentration in the A375R sample but high in both A375 and A375R + NCTD samples. Heatmaps of significantly changed metabolic concentration were produced using the R package “gplots” function named heatmap.2() (Warnes et al., 2005). The metabolic pathway enrichment analysis was used by Metaboanalyst 5.0 (https://www.metaboanalyst.ca/).

### Analysis of the transcriptome data

We analyzed the transcriptome data to highlight differential gene expression associated with the application of NCTD to the cell lines. RNA-seq reads from each library were mapped to the human reference genome using “HISAT2” (Kim et al., 2019). Gene expression levels were quantified using “HTSeq” (Anders et al., 2015). The differential gene expression analysis was performed by “edgeR” (Robinson et al., 2010). Volcano plots of genes were generated with log-fold-change values and adjusted *p* values obtained by the R package “EnhancedVolcano” (https://github.com/kevinblighe/EnhancedVolcano) based on edgeR statistical analysis results. Downregulation of a gene is defined by a high expression level in A375R samples but low in both A375 and A375R + NCTD samples. Upregulation of a gene is defined by a low expression level in the A375R sample but high in both A375 and A375R + NCTD samples. Heatmaps of significant differential gene expression were produced using the R package “gplots” function named heatmap.2() (Warnes et al., 2005). Significantly different genes were subjected to the KEGG pathway enrichment analysis using the David website (http://david.ncifcrf.gov/).

### Statistical analysis

All experimental analysis was represented as the mean ± standard deviation (SD) from three times of replicative experiments. Microsoft Excel and GraphPad Prism 5 were used to draw the charts. The *p*-value of the two groups was compared using the Student’s *t*-test and the one-way analysis of variance using more than two groups. A value of *p* < 0.05 was used to indicate a statistically significant difference.

## Results

### Norcantharidin displays significant proliferation inhibitory effect in A375R cells

**FIGURE 1:**
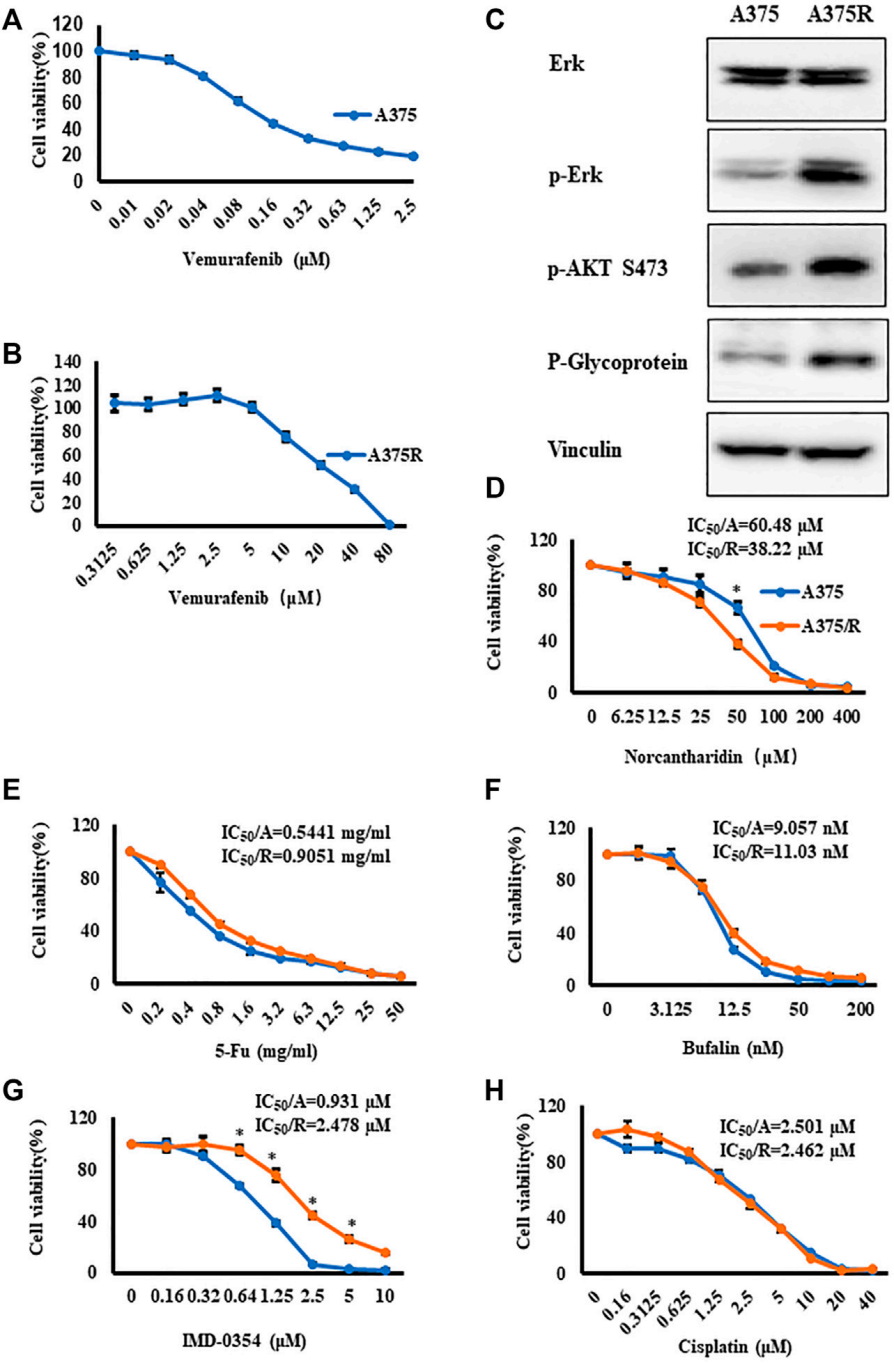
Vem-resistant melanoma cells (A375R) were generated, and NCTD displayed potent effect on A375R. **(A,B)** A375 **(A)** and A375R **(B)** were treated with indicated concentrations of Vem for 48 h, cell viability was measured by CCK8 assays. **(C)** Erk, p-Erk, p-AKT S473, and P-glycoprotein in A375 and A375R were detected by Western blot analysis. **(D–H)** Cisplatin **(D)**, 5-Fu **(E)**, bufalin **(F)**, IMD-0354 **(G)**, and NCTD **(H)** were cultured with A375 and A375R for 48 h, and cell viability was measured by CCK8 assays. Data were shown as mean ± SD from three independent experiments, ∗*p* < 0.05.

First, A375 cells were treated with Vem up to 2.5 μM for 48 h, and CCK8 assays showed that IC_50_ of Vem was 0.157 μM (Figure 1A). To establish Vem-resistant melanoma cell lines (A375R), we passaged A375 cells in the presence of Vem from 0.05 μM gradually up to 10 μM. Vem-resistant cell lines (A375R) displayed an IC_50_ value of 21.5 μM, 100-fold over that of the parental line (A375) (Figure 1B). ERK and PI3K-AKT pathways as well as P-glycoprotein, well-known hallmarks of acquired Vem-resistant cells, were significantly upregulated in A375R compared to A375 (Figure 1C). These results consisted of previous studies that verified the resistance of melanoma cells against BRAF inhibitors (Luebker and Koepsell, 2019).

We proposed to use several antitumor agents that promised to alter cancer metabolism to overcome the Vem resistance, such as cisplatin (Liang et al., 2008), 5-Fluorouracil (5-Fu) (Zhao et al., 2014), bufalin (Li et al., 2018), IMD-0354 (Feng et al., 2021), and norcantharidin (NCTD) (Zhang et al., 2020). Thereafter, the cytotoxicity of these anticancer agents was evaluated in A375R cells and A375 cells. CCK8 assay results showed that except for NCTD and IMD-0354, all agents displayed identical cytotoxicity between two cell lines (Figures 1E,F,H). A375R also developed resistance to IMD-0354 (Figure 1G). Intriguingly, NCTD exhibited significantly higher cytotoxicity against A375R cells compared to A375 cells (Figure 1D). The IC_50_ values of the aforementioned medicines were shown in Figures 1D–H. These results prompted that NCTD might target the signaling pathway that accounted for the resistance development.

### Norcantharidin induces cell cycle arrest in the G2/M phase of A375R cells

**FIGURE 2:**
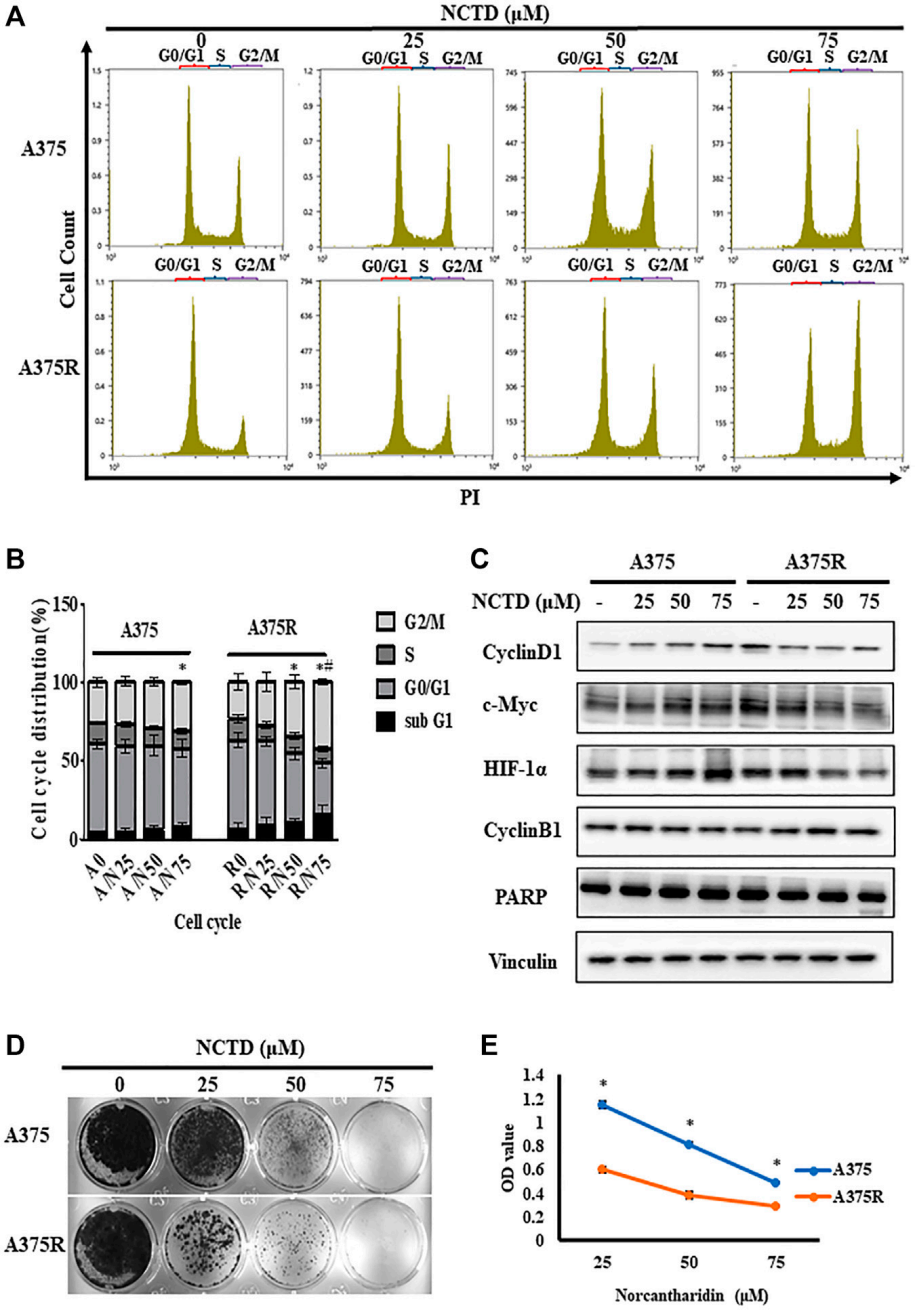
NCTD induced cell cycle arrest in the G2/M phase in A375R. **(A,B)** Distribution of cell cycle for A375 and A375R cells after being treated with various concentrations of NCTD for 12 h. **(C)** A375 and A375R cells after being treated with various concentrations of NCTD for 12 h, and Cyclin D1, Cyclin B1, c-Myc, HIF-1α, PARP, and Vinculin were detected by Western blot. **(D)** Represented photographs showed different concentrations of NCTD-inhibited A375 and A375R cells for a long period of 7 days using the crystal violet staining method. **(E)** Supernatant medium of A375 and A375R cells treated with NCTD was recultivated and continued to culture for 48 h, and the cell viability was then measured by CCK8 assay. The DNA contents of cells at different cell phases were presented as the mean ± SD of three independent experiments. The statistically significant difference was labeled as ∗when *p* < 0.05 in the same cell group and ^#^ when *p* < 0.05 between control in different cell groups.

Previous studies demonstrated that NCTD could induce the G2/M arrest in osteosarcoma (Zhu et al., 2019), therefore, we investigated the differences in cell cycle distribution between A375 and A375R treated with NCTD by flow cytometry. As shown in Figures 2A,B, the percentage of the G2/M phase significantly increased in A375R cells after 24 h of the NCTD treatment in a concentration-dependent manner. Especially, the number of A375R cells in the G2/M phase increased from 23.53% to 42.7% following the NCTD treatment (75 μM), whereas A375 cells showed slight G2/M accumulation (from 25.6% to 30.92%) (Figure 2B). It is well-known that the cell cycle is regulated by various proteins including cyclins and their kinase partners-cyclin dependent kinase (CDKs). Among those, Cyclin B1 is the predominant regulator in the G2/M phase (Wang et al., 2014). In A375R cells, 12 h of the NCTD treatment obviously increased the Cyclin B1 expression and decreased the levels of c-Myc, HIF-1α, and Cyclin D1 in concentration-dependent manners (Figure 2C; Supplementary Figure S2). Moreover, apoptosis was also determined in both two cell lines treated with NCTD. However, there was no remarkable subG1 peak that appeared in both A375R cells and A375 cells following the NCTD treatment during the cell cycle analysis (Figures 2A,B). To further quantify apoptotic cells, we stained NCTD-treated cells with Annexin V-PI and analyzed them with flow cytometry. The NCTD treatment did not significantly increase apoptotic A375R cells in dose-dependent manner, but the apoptosis rate was 10.68% after treatment with 75 μM (Supplementary Figures S1A,B). Consistently, the cleavage of PARP, a key surrogate marker for apoptosis, was also barely detectable in both two cell lines after the NCTD treatment, only if A375R cells were treated with 75 μM, cleaved PARP were detected (Figure 2C; Supplementary Figure S2). These findings indicated that NCTD resulted in the G2/M phase arrest in A375R cells but was followed by no apparent apoptosis.

Furthermore, we analyzed the fate of A375 cells treated with NCTD. Colony formation assays unveiled that NCTD exhibited a more proliferation inhibitory effect in A375R cells than that in A375 cells (Figure 2D). Moreover, after A375 and A375R cells were treated with different concentrations of NCTD for 48 h, the supernatants containing numerous suspending cells were collected and transferred to a new 96-well plate with a density of 1 × 10^4^ cells per well, then the suspending cells were further cultured for another 48 h. Of note, NCTD-induced suspending cells were still partially able to adhere. Nevertheless, the number of adherent A375R was significantly lower than that of A375, indicating higher sensitivity of A375R to NCTD compared to A375 (Figure 2E). These results suggested that the proliferation of both adherent and suspending A375R treated with NCTD were inhibited by G2/M-arrest, while others partly die by other means.

### Norcantharidin inhibits Vem-induced upregulation of the pentose phosphate pathway

**FIGURE 3:**
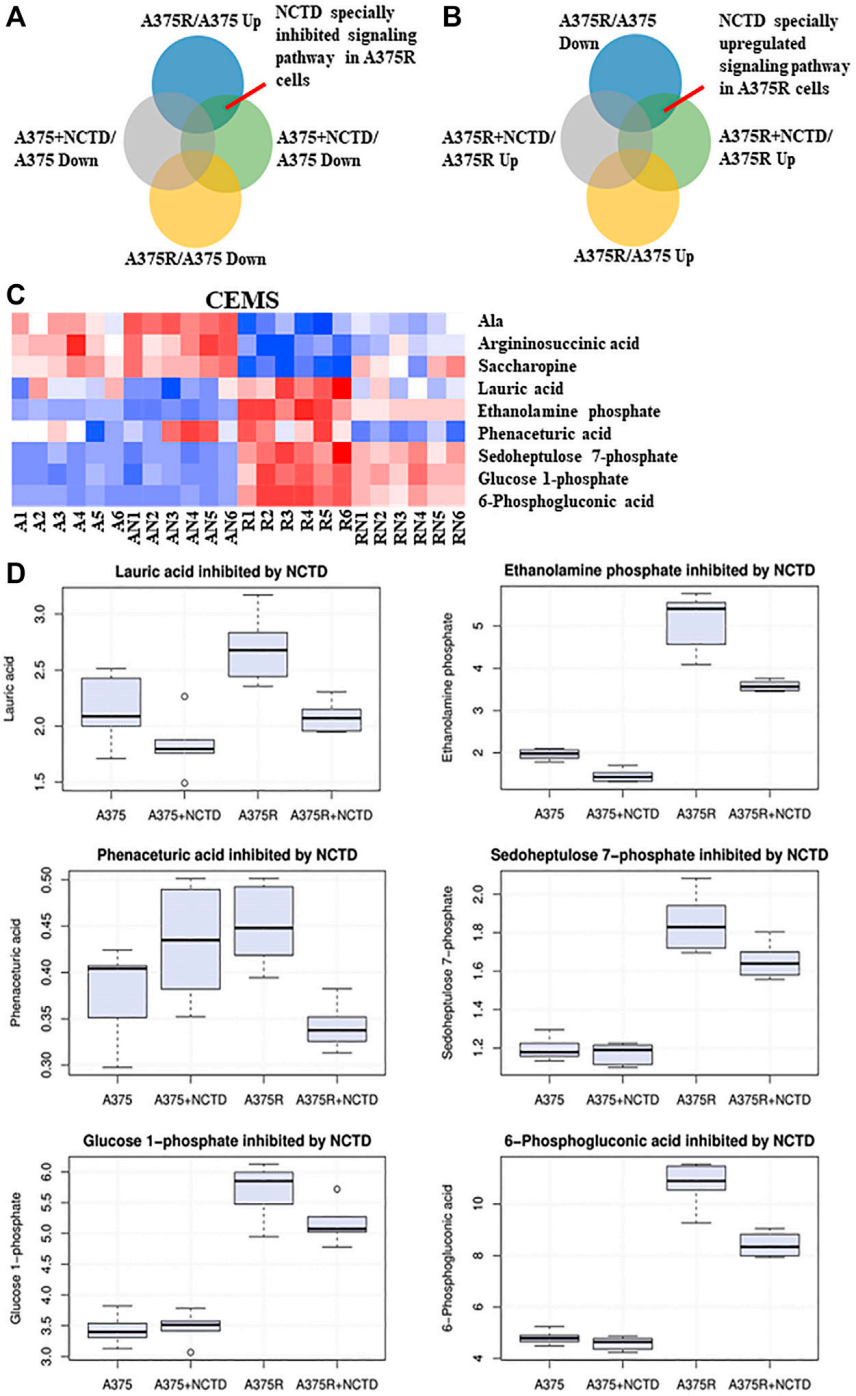
NCTD inhibited Vem-induced upregulation of PPP based on CE-MS metabolomics analysis. **(A,B)** Intersection of many sets of metabolites revealed, especially downregulating and upregulating metabolites of NCTD in A375R cells. **(C)** Heatmap of significantly changed metabolites based on CE-MS in the A375, A375R, A375 + NCTD, and A375R + NCTD samples. Red in the heatmap represented the upregulated metabolites, and blue represented downregulated metabolites. **(D)** Six histograms showed statistically significantly downregulated metabolites in A375R cells.

**FIGURE 4:**
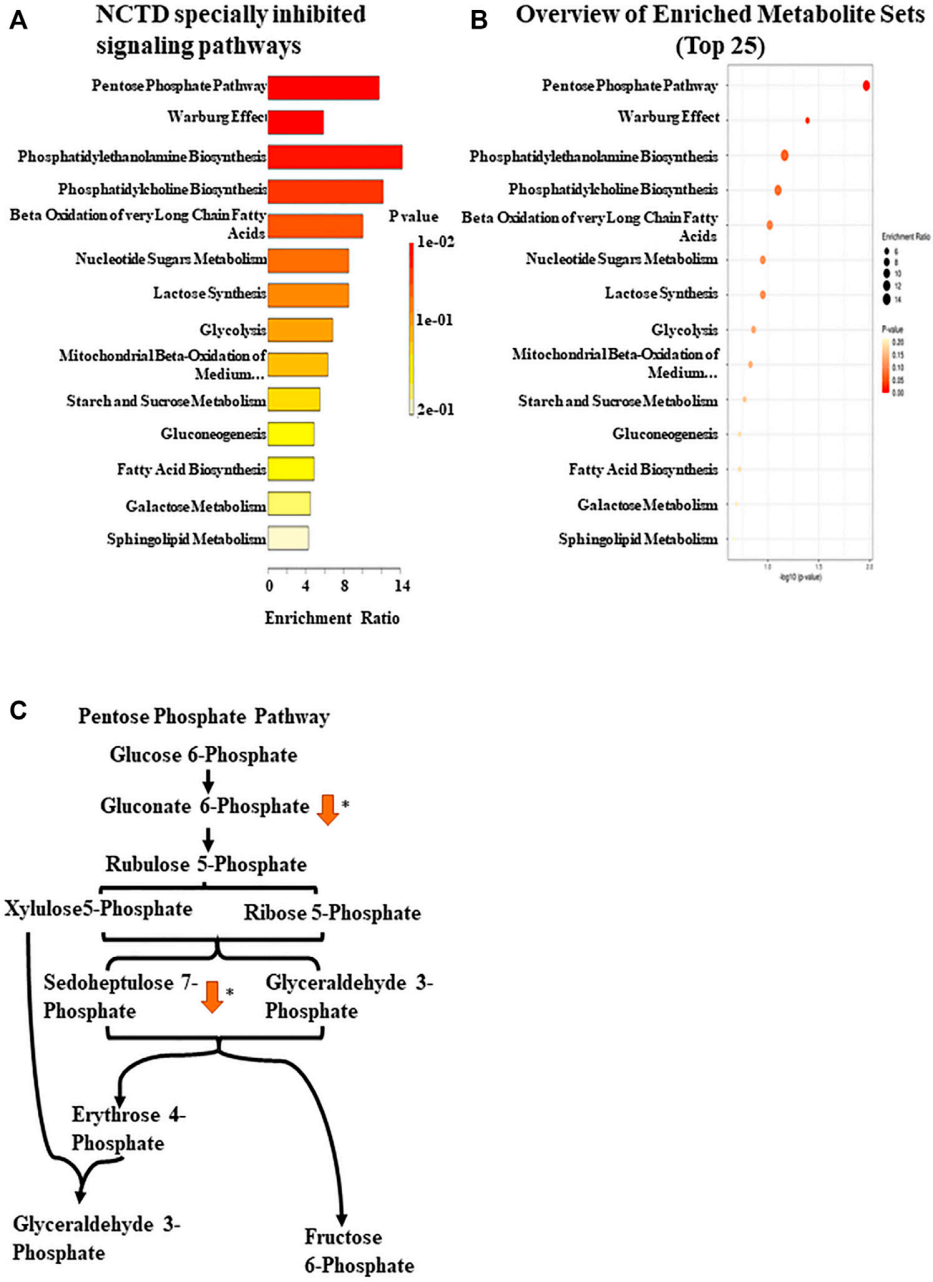
Enrichment pathway analysis based on CE-MS. **(A)** Metabolic pathway enrichment analysis of the metabolites inhibited by NCTD in A375R cells was used by Metaboanalyst 5.0 (https://www.metaboanalyst.ca/). **(B)** Enrichment ratio of the metabolic pathway. **(C)** Regulatory mechanism of the pentose phosphate pathway by NCTD. ∗*p* < 0.05.

BRAF-mutated melanoma is known to possess high glycolytic activity (Parmenter et al., 2014). Metabolomics has emerged as a powerful tool for the discovery of biomarkers in various cancer types, and some studies have assessed metabolic alterations in Vem-resistant melanoma (Baenke et al., 2016). Previously, our preliminary study found that NCTD could lead to metabolic changes in multiple cell lines, which has not been published yet. To further explore the mechanisms of NCTD against Vem-resistant melanoma, we used CE-MS and LC-MS to compare metabolic profiling between two cell lines with or without the NCTD treatment. We extracted metabolites from three metabolic data sets including “A375R versus A375,” “A375R with NCTD versus A375R,” and “A375 with NCTD versus A375” (Figures 3A,B). As shown in Figures 3C,D, the lauric acid, ethanolamine phosphate, phenaceturic acid, sedoheptulose 7-phosphate, glucose 1-phosphate, and 6-phosphogluconic acid, were remarkably increased in A375R cells compared to A375 cells, which was significantly reduced following the NCTD treatment. Among such metabolites, sedoheptulose 7-phosphate and 6-phosphogluconic acid are the critical metabolites involved in PPP (Figure 4C). According to the enrichment analysis of metabolic pathways (Figures 4A,B), PPP was specially downregulated in NCTD-treated A375R cells (*p* < 0.05). These results revealed that NCTD exhibited metabolic modulatory effects on Vem-resistant BRAF V600E-mutated melanoma *via* reversing Vem-induced PPP upregulation.

### Norcantharidin targets Vem-induced lipid alterations

**FIGURE 5:**
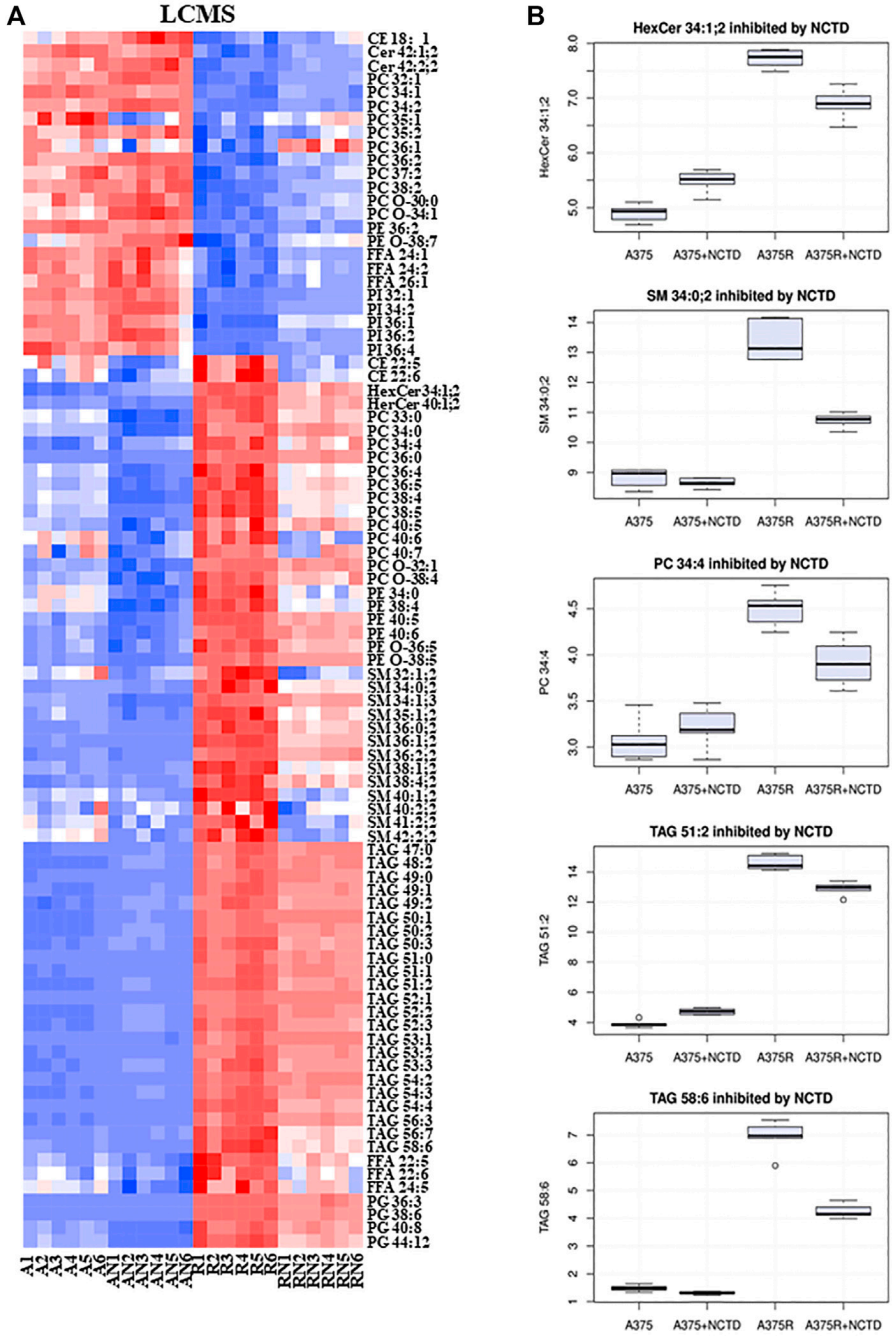
NCTD targeted Vem-induced lipid alterations based on the LC-MS metabolomics analysis. **(A)** Heatmap of significantly changed lipids based on LC-MS in the A375, A375R, A375 + NCTD, and A375R + NCTD samples. Red in the heatmap represents the upregulated lipids, and blue represents downregulated lipids. **(B)** Some histograms showed statistically significantly downregulated lipids in A375R cells.

In many types of cancer, lipid metabolism supports cancer cell growth and metastasis as a prominent pathway for energy storage and supply. We used LC-MS to explore the lipid alterations in A375 cells and A375R cells with or without the NCTD treatment. Figures 5A,B summarized the data on lipidomic profiles of all groups. We also extracted metabolites from three metabolic data sets including “A375R versus A375,” “A375R with NCTD versus A375R,” and “A375 with NCTD versus A375” (Figures 3A,B), and 115 key lipid metabolites were identified. The most significantly altered lipids were triacylglycerols (TAGs), sphingomyelins (SMs), phosphatidylglycerols (PGs), hexosylceramides (Hex-Cers), Ceramindes (Cers), and cholesterol esters (CEs). Most TAGs, SMs, PGs, Hex-Cers, and CEs were significantly upregulated in A375R cells, whereas downregulated in NCTD-treated A375R cells. Additionally, phosphatidylcholine (PCs) including 33:0, 34:0, 34:4, 36:0, 36:4, 36:5, 38:4, 38:5, 40:4, 40:6, 40:7, O-32:1, O-38:4, and FFAs (22:6, 22:5, 24:5) were also downregulated by NCTD in A375R cells. Conversely, several metabolites including Cers (42:1; 2, 42:2; 2), PIs (32:1, 34:2, 36:1, 36:2, 36:4), PCs (32:1,34:1, 34:2, 35:1, 35:2, 36:1, 36:2, 37:2, 38:2, O-30:0, O-34:1), PEs (36:2, O-38:7), and FFAs (24:1, 24:2, 26:1) were decreased in A375R cells but increased following the NCTD treatment. These results indicate that the Vem resistance resulted in alterations of lipid metabolism and NCTD could target such alterations.

### The network of transcriptomics and metabolomics to overcome Vem-resistant melanoma by norcantharidin

**FIGURE 6:**
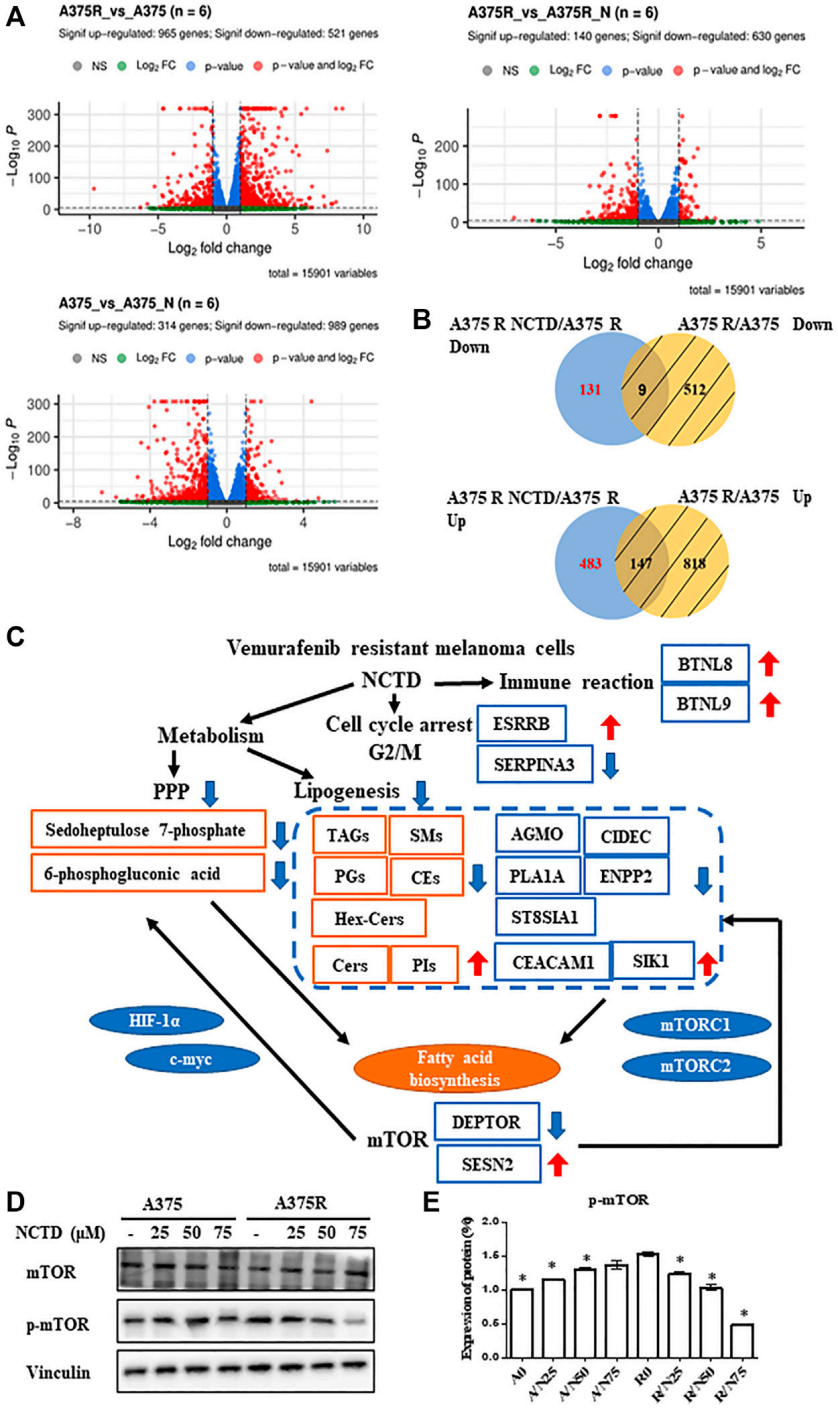
Integrated transcriptomics and lipidomics network analysis. **(A)** Volcano plot of differentially expressed genes in A375R versus A375, A375 versus A375 + NCTD, and A375R versus A375 + NCTD groups. **(B)** Intersection of many sets of genes revealed, especially inhibiting and upregulating genes of NCTD in A375R cells. **(C)** Differential correlation of metabolites, lipids, and genes. **(D)** A375 and A375R cells after being treated with various concentrations of NCTD for 12 h, and mTOR and p-mTOR were detected by Western blot. **(E)** Expression of the protein in Western blotting was evaluated as a fold of vinculin. Data were shown as mean ± SD from three independent experiments.

To further explore NCTD-induced alterations in the cellular signaling pathway, an unbiased RNAseq analysis was performed on A375 and A375R cells after they were, respectively, subjected to IC_50_ (60 and 38 μM) of NCTD for 24 h period. The volcano plots of three groups showed that 965 genes were upregulated and 521 genes were downregulated in the A375R versus A375 group; meanwhile, 140 genes increased and 630 genes decreased in the A375R versus A375R + NCTD group, and 314 genes increased and 989 genes decreased in the A375 versus A375 + NCTD group (Figure 6A). Since the analysis was carried out on specific conditions (the genes were specially modulated by NCTD), we excluded genes altered by the Vem resistance and extracted the differentially expressed genes, which included the upregulation of genes (*n* = 483) and downregulation of genes (*n* = 131) (Figure 6B). Among these genes, five downregulated genes including AGMO, CIDEC, PLA1A, ENPP2, and ST8SIA1 as well as two upregulated genes including CEACAM1 and SIK1, which were significantly modulated by NCTD in A375R, had a close regulatory relationship with lipid metabolism (Figure 6C). It has been reported that CEACAM1 acts the negative regulatory roles in the fatty acid biosynthetic process (Russo et al., 2016), and SIK1 exhibits suppression of the triglyceride biosynthetic process (D et al., 2020). Activation of mTORC1 usually causes significant upregulation of oxidative PPP and promotes the synthesis of the lipid phosphatidylcholine (Porstmann et al., 2008). Interestingly, several altered genes induced by NCTD in A375R cells are closely associated with the mTOR signaling pathway (Figure 6C). For example, inhibiting ST8SIA1 can sensitize triple-negative breast cancer to chemotherapy *via* suppressing FAK/AKT/mTOR (Wan et al., 2021). CIDEC was decreased by Allulose in aged mice, which was regulated by mTOR (Kim et al., 2022). ENPP2 mediates enhanced mTOR signaling in chondrogenic induction of fibrodysplasia ossificans progressive-mesenchymal stromal cells (FOP-iMSCs) (Hino et al., 2017). The suppression of the SIK1/P53 signaling pathway enhanced SIK3/mTOR signaling and potentiated aerobic glycolysis-mediated cell growth in breast cancer cells (Ponnusamy and Manoharan, 2021). Moreover, the gene level of DEPTOR was downregulated and SESN2 was upregulated by NCTD in A375R cells (Figure 6C). Of note, DEPTOR has been documented to play important roles in triglyceride/free fatty acid (TG/FFA) circling and fatty acid synthesis, which is mediated by mTOR pathway activation (Caron et al., 2015). SESN2 plays a negative regulatory role in amino acids (AA)-mediated cell proliferation and synthesis of casein *via* downregulating the mTORC1 pathway (Xu et al., 2015). Taken together, according to the integration of metabolome and transcriptome analysis, we found that the mTOR pathway was significantly downregulated by NCTD in Vem-resistant melanoma cells, and validated these results by detecting the protein expression. Consistently, phosphorylation of mTOR was decreased by NCTD in A375R cells (Figures 6D,E), and still no obvious alteration in A375 cells.

Meanwhile, the downregulation of SERPINA3 and upregulation of ESRRB by the NCTD treatment are found to be able to cause cell cycle arrest in the G2/M phase (Yang et al., 2014; Tiek et al., 2019). Additionally, based on the enrichment analysis in the Reactome pathway, we found that BTN family genes, such as BTNL8 and BTNL9, which play a vital role in the immune signaling pathway, were also obviously upregulated by NCTD in Vem-resistant melanoma. These findings indicate that in addition to regulating cell metabolism and cell cycle in melanoma, NCTD might also play key roles in modulating the T-cell response.

## Discussion

Melanoma is the least general type of skin cancer that is caused by mutations in multiple genes, along which V600E mutant represents 70%–90%, resulting in the most deaths of skin cancer (Hodis et al., 2012). Moreover, Vem, a promising drug targeting BRAF V600E mutation, is compromised by acquired resistance in V600E melanoma. Thus, uncovering the mechanism underlying resistance development and discovering effective therapies to resolve resistance is still the primary concern in clinics.

Targeting the MAPK/ERK pathway with MEK inhibitors improves overall survival (McArthur et al., 2014; A et al., 2012; Long et al., 2015), but acquired resistance is almost inevitable. A recent report has suggested that targeting mTOR signaling overcame acquired resistance in BRAF-mutant melanoma (Wang et al., 2021), implying targeting signaling pathways bypass MAPK/MEK signaling to bring out great improvement. In line with this, NCTD also downregulated mTOR activation in A375R. However, results indicated that the mTOR pathway is not the intrinsic target of NCTD, since NCTD showed no significant inhibitory effect on mTOR in parental A375 cells. Modulation of other pathway cross talk mTOR pathways by NCTD in A375R cells might impair cell proliferation and growth. By conducting an integrated analysis of the complex network of transcriptomics and metabolomics, we speculated that NCTD modulated cell metabolism and thereafter destructed A375R reestablished cellular metabolism.

The mTOR pathway plays a major role in the regulation of protein translation, cell growth, and metabolism. It also responds to alteration in metabolites. Here, inhibition of the mTOR pathway also reflected that NCTD perturbed A375R metabolic homeostasis. Previous studies found that NCTD could induce alterations in lipid metabolism of hepatocytes (Zhao et al., 2019), and could also suppress glycolysis in colorectal cancer cells *via* Fam46c and inhibit ERK1/2 signaling (Zhang et al., 2020). However, the question of whether this metabolic modulatory effect of NCTD is applicable to drug-resistance cells has not been asked yet. Metabolic reprogramming is very common in cancer development, which also can be seen in drug-resistance cells. For example, Vem-resistant melanoma cells indeed changed their metabolism and exhibited a higher degree of reliance on lipid metabolism (Delgado-Goñi et al., 2020; Chang et al., 2022). Our results showed that NCTD affected PPP and lipid metabolism, which was the plausible mechanism of its anti-proliferation effect on Vem-resistant melanoma. Most TAGs, SMs, Hex-Cers, and CEs increased in Vem-resistant melanoma cells and were downregulated by the NCTD treatment. TAGs and CEs are important to melanoma cell progression (Du et al., 2020). SM is a type of sphingolipid accounting for 85% of all sphingolipids in the cell membrane (Kolesnick, 1994), and ensures sufficient material for membrane construction to meet with fast proliferating cancer cells. Ceramide belongs to a part of phospholipid which was considered as a controlling factor for cancer inhibitor, inducing cell cycle arrest, and apoptosis (Pettus et al., 2002). Here, by applying NCTD, we report a new promising agent to overcome Vem-resistance by disrupting metabolic homeostasis in Vem-resistance cells. By conducting transcriptomics and metabolomics, we revealed PPP, lipid metabolism, and mTOR signaling is involved in the NCTD pharmacological action. However, the regulation and function of mTOR and its cross talk with the metabolic pathway is a highly complex and dynamic process. Some studies have shown that the mTOR pathway played a crucial role in regulating the processes of cell proliferation as well as lipid biosynthesis (Caron et al., 2015). Activation of mTORC1 usually causes significant upregulation of oxidative PPP and promotes the synthesis of the lipid phosphatidylcholine (Porstmann et al., 2008). Accordingly, the NCTD treatment simultaneously downregulated PPP, lipogenesis, and mTOR activation, suggesting these changes in metabolic pathways might result from mTOR inactivation. In addition, Myc and HIF-1α-mediated glutaminolysis cross talk in lipogenesis and cholesterol synthesis are synthesized (Koppenol et al., 2011; Vaupel et al., 2019). NCTD suppressed c-Myc and HIF-1α expression in Vem-resistant cells, which also possibly resulted in the downregulation of lipogenesis in Vem-resistant cells. However, the transcriptomics analysis showed that mTORC1-related genes DEPTOR was downregulated, and SESN2 was upregulated by the NCTD treatment, which could subsequently lead to mTORC1 inactivation. These changes shown in transcriptomics might attribute to the NCTD-induced metabolic alteration. Most of the results indicated that NCTD showed different effects on cell metabolism and cell proliferation-related signaling pathways in two cell lines. We reasoned that these different phenotypes such as downregulation of PPP, lipid metabolism, and mTOR signaling might be the mechanism of action of NCTD. In fact, hidden targets still remain to be unconcealed by conducting an intensive study or temporal omics to confirm the relation between downregulation of PPP as well as lipid metabolism and mTOR signaling inhibition, or to trace back the precise target of NCTD.

BTN, one type of T-cell receptors (TCRs), can modulate the response of T-cells and further affect tumorigenesis and cancer progression (Melandri et al., 2018). BTNL2, BTNL3, BTNL8, BTNL9, and SKINTL constitute the BTNL family, which regard as a tumor inhibitor in many cancers, such as malignant melanoma and breast cancer. It has been reported that BTNL8 may be important for co-stimulation of primary immune response in the periphery at sites of inflammation, and BTNL9 can inhibit cancer aggression in melanoma cells (Jiang and Liu, 2019). Therefore, regulating BTNL8 and BTNL9 might be a potential anticancer mechanism of NCTD, which could be further investigated to bring out a new strategy for anti-resistance therapy.

In conclusion, the present study demonstrated that NCTD displayed a significant cancer cells proliferation inhibitory effect and induced the G2/M cycle arrest in Vem-resistance melanoma. Furthermore, the upregulation of the pentose phosphate pathway and lipogenesis were reversed by the NCTD treatment possibly *via* the mTOR pathway in Vem-resistance melanoma. Targeting metabolic pathways and the mTOR pathway may facilitate the development of new therapies to overcome Vem-resistance problems in BRAF mutated melanoma.

## Data Availability

All datasets generated for this study are included in the article. The transcriptomics data presented in the study are deposited in the NCBI-Sequence Read Archive,with the accession number PRJNA855020 (https://www.ncbi.nlm.nih.gov/bioproject/PRJNA855020).
