# Supplementary material for: Norcantharidin overcomes vemurafenib resistance in melanoma by inhibiting pentose phosphate pathway and lipogenesis via downregulating the mTOR pathway
Source: Front Pharmacol. 2022 Aug 12;13:906043. doi: 10.3389/fphar.2022.906043 (PMC9411668; doi:10.3389/fphar.2022.906043)
Supplement: Supplementary file 1 [file DataSheet1.docx]

**Supplementary**

**Norcantharidin overcomes vemurafenib resistance in melanoma by inhibiting pentose phosphate pathway and lipogenesis via downregulating mTOR pathway**

**
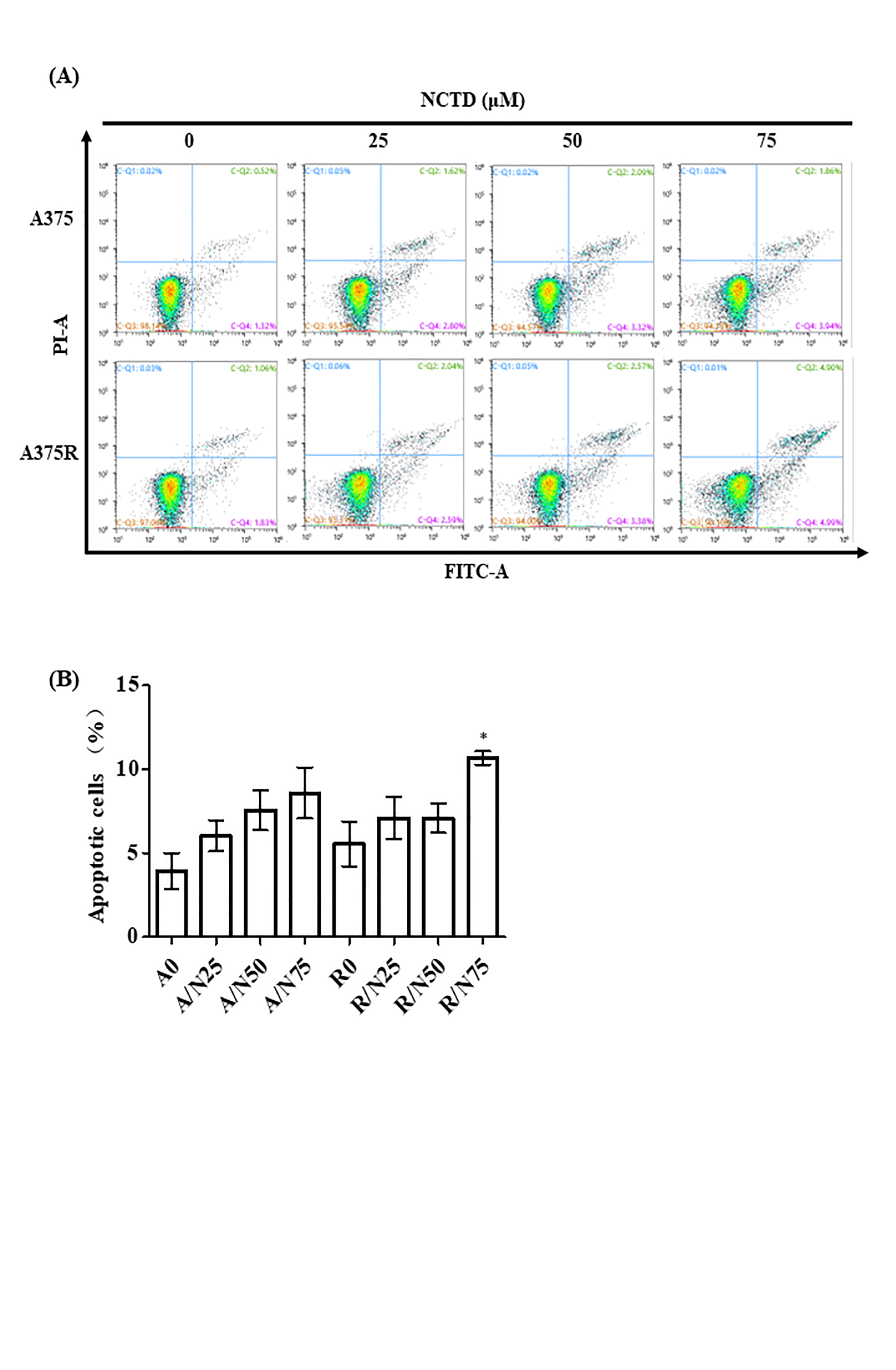
**

**Figure S1**

**Apoptosis analysis of NCTD. (A, B)** Distribution of apoptosis rates for A375 and A375R cells after treated with various concentrations of NCTD for 24 hours. The apoptosis analysis was evaluated using the Annexin V-FITC/PI Apoptosis Detection by a flow Cell Sorter. The statistically significant difference was labeled as *when *P＜0.05* in the same cells group and #when *P＜0.05* between control in different cells groups.

**
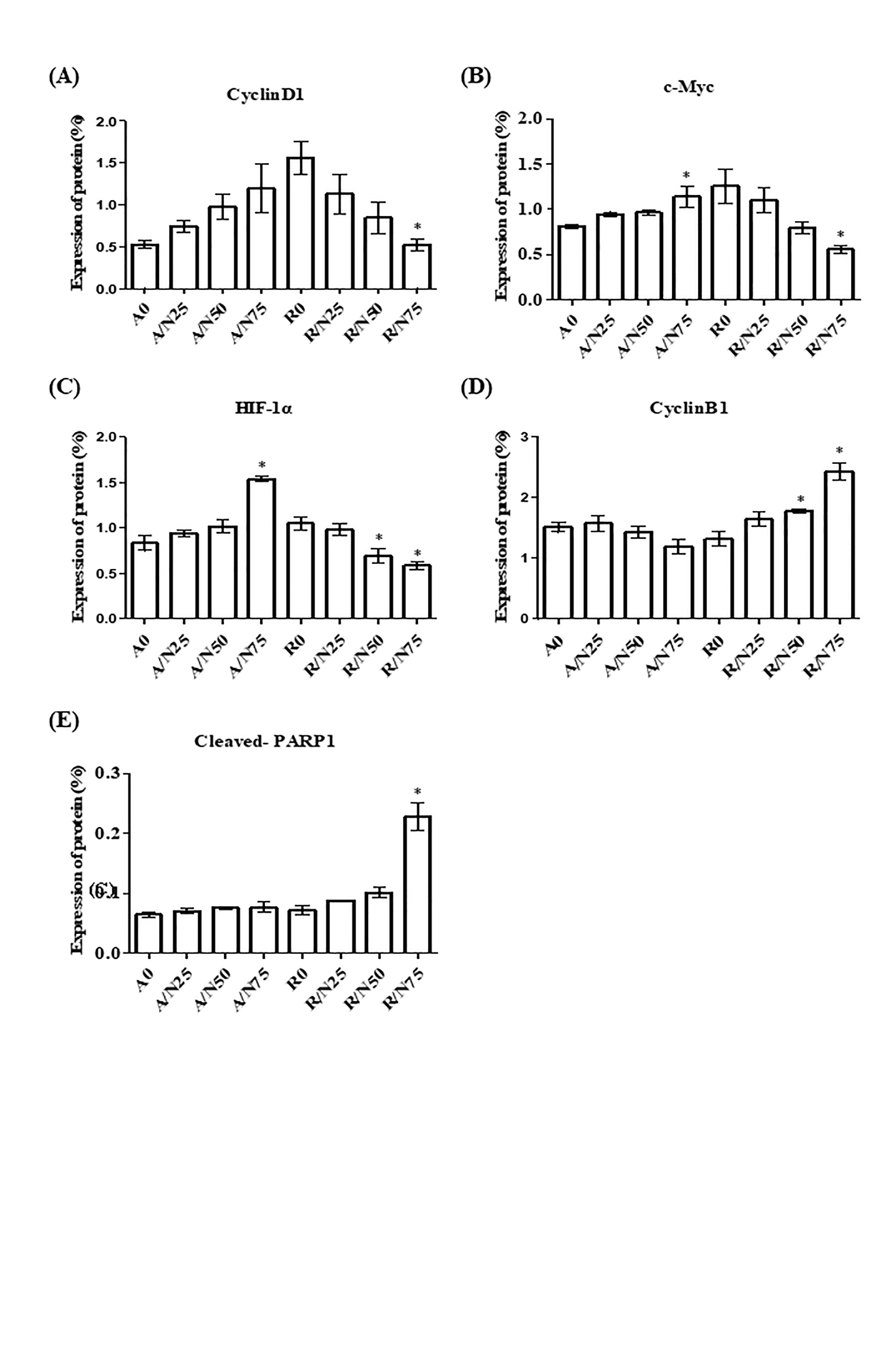
**

**Figure S2**

**The expression of protein in western blotting.** (A, B, C, D, E) The expression of protein in western blotting was evaluated as a fold of vinculin. Data were shown as mean ± SD from three independent experiments. The statistically significant difference was labeled as *when *P＜0.05* in the same cells group.
